# 1020. BioFiring on all Cylinders: Validation of BioFire FilmArray Pneumonia Panel and Determination of Optimal Utility

**DOI:** 10.1093/ofid/ofab466.1214

**Published:** 2021-12-04

**Authors:** Rachel Strength, Jessica Bennett, Jay Patel, Jarred Bowden, Jane V Eason, Neena Thomas-Gosain

**Affiliations:** 1 University of Tennessee Health Science Center, Memphis, Tennessee; 2 VAMC Memphis, Germantown, Tennessee; 3 University of Tennessee Health Sciences Center, Memphis, Tennessee; 4 Memphis VA Medical Center, Memphis, Tennessee; 5 Memphis VA Medical Center; UTHSC, Memphis, Tennessee

## Abstract

**Background:**

Respiratory cultures can take up to five days to grow, time that can be crucial in treating patients with serious infections. Newer rapid microbiological identification tests are designed to shorten this delay between specimen collection and test result. The BioFire^®^ FilmArray^®^ Pneumonia Panel is a multiplex PCR panel that can identify 8 viral, 18 bacterial, and 7 resistance gene targets in one hour. In this study, we aimed to calculate the predictive value of this test and its utility in the clinical setting.

**Methods:**

This retrospective study compared BioFire® FilmArray® Pneumonia Panel results to respiratory cultures run at our center from 3/1/2020 to 2/28/2021. For every BioFire sample, a respiratory culture was run concurrently. We examined correlations between these two tests using data collected from the microbiology laboratory and the electronic medical record.

**Results:**

190 BioFire samples from 124 patients were submitted for processing. Of these, 148 samples had a concomitant respiratory culture result that grew organisms that BioFire could detect. BioFire and culture results were compared, and sensitivity and specificity were calculated on a per-sample basis. Sensitivity was calculated at 91%, specificity at 67%, positive predictive value at 46%, and negative predictive value at 96%.

BioFire detected 30 resistance genes total, including *mec*A/C and MREJ, CTX-M, and KPC. The sensitivity and negative predictive value for BioFire resistance gene detection was 100%. However, specificity was 94-98%, and the positive predictive value ranged between 25-41% when compared to culture.

**Conclusion:**

Despite the promise of faster results and better screening, our data suggests that further study is needed to determine the utility of the BioFire pneumonia panel. The strength of the panel appears to lie in its negative predictive value and sensitivity, but as a positive predictive tool, it is suboptimal.

**Disclosures:**

**All Authors**: No reported disclosures

